# The Effects of Baicalin on Myoglobinuric Acute Renal Failure in Rats

**DOI:** 10.4274/balkanmedj.2017.0040

**Published:** 2018-01-20

**Authors:** Özlem Yalçınkaya Yavuz, Nurettin Aydoğdu, Ebru Taştekin, Necdet Süt

**Affiliations:** 1 Department of Physiology, Trakya University School of Medicine, Edirne, Turkey; 2 Department of Pathology, Trakya University School of Medicine, Edirne, Turkey; 3 Department of Biostatistics, Trakya University School of Medicine, Edirne, Turkey

**Keywords:** Baicalin, acute kidney injury, free radicals, nitric oxide, nitric oxide synthase

## Abstract

**Background:**

Myoglobinuric acute kidney injury is a uremic syndrome that develops due to damage of skeletal muscle. Free radicals and nitric oxide play an important role in the pathogenesis of myoglobinuric acute kidney injury. Baicalin has multiple bioactivities, including antimicrobial, anti-inflammatory and antioxidant properties and is a potent free radical scavenger.

**Aims:**

To investigate the nephroprotective mechanism of baicalin on myoglobinuric acute kidney injury.

**Study Design:**

Animal experimentation.

**Methods:**

In our study, male Sprague Dawley rats were divided into 4 groups. Control (n=8), Baicalin (n=8), myoglobinuric acute kidney injury (n=10) and myoglobinuric acute kidney injury + baicalin (n=10). The rats were deprived of water for 24 hours before receiving intramuscular injection. The control and baicalin groups were injected intramuscularly with saline (8 ml/kg), and the myoglobinuric acute kidney injury and myoglobinuric acute kidney injury + baicalin groups were given 50% glycerol 8 ml/kg. One hour later, the control and myoglobinuric acute kidney injury groups received saline intraperitoneally, and the baicalin and myoglobinuric acute kidney injury + baicalin groups were given 200 mg/kg baicalin. Twenty-four hours after the glycerol injection, urine and blood samples were taken, and the kidneys of the rats were harvested under intraperitoneally injections of anaesthesia.

**Results:**

We found that the levels of creatinine, urea, nitric oxide, alanine transaminase, aspartate aminotransferase, creatine kinase in serum samples, malondialdehyde, nitric oxide, inducible nitric oxide synthase, and endothelial nitric oxide synthase concentrations in renal tissue were increased in the myoglobinuric acute kidney injury group compared with the control group (p<0.05). The nitric oxide and glutathione levels in the kidney were significantly decreased in the myoglobinuric acute kidney injury + baicalin group compared with the myoglobinuric acute kidney injury group (p<0.05). There were no significant differences between any other parameters.

**Conclusion:**

Our results did not show any protective effect of baicalin on myoglobinuric acute kidney injury, possibly because the different effective factors in the pathogenesis of experimental myoglobinuric acute kidney injury used in this experiment deviate from other experimental models. Moreover, detailed studies are needed to clarify the effects of baicalin in different doses and treatment durations in glycerol-induced acute kidney injury model.

Acute kidney injury (AKI) is a common clinical syndrome characterised by sudden reduction in renal function that develops due to local and systemic causes and affects all systems (1). Rhabdomyolysis is a syndrome that occurs due to traumatic or non-traumatic muscle injury. Contents of damaged skeletal muscle cells enter the circulation and cause clinical and laboratory findings (2,3).

Mine collapses, traffic accidents, wars, natural disasters and trauma linked to earthquakes are among the most important causes of rhabdomyolysis. Apart from trauma, rhabdomyolysis can be caused by alcohol, illegal drugs and lipid-reducing agents. In the USA, there are nearly 26.000 rhabdomyolysis cases observed each year and statistics indicate that alcohol and drugs are responsible for 81% of all cases (2,4). One of the major causes of death after earthquakes is myoglobinuric acute kidney injury (MAKI) induced by rhabdomyolysis. For this reason, preventative medications for MAKI have vital importance (5). The experimental model of MAKI is induced by intramuscular injections of 50% hypertonic glycerol in rats. This model is accepted as equivalent to myoglobinuric AKI developing in humans (6). Intramuscular injection of hypertonic glycerol causes severe muscle damage. This results in intravascular volume reduction and vasoconstrictor endotoxic cytokine release. After severe muscle damage, large amounts of HEM protein enter the circulation due to myolysis and hemolysis. Binding of HEM proteins to nitric oxide (NO), a potent vasodilator, causes strong contractions in the renal vasculature. This results in reduced renal function, increased oxidative stress, reduced NO levels and histopathological kidney damage (7,8).

After skeletal muscle damage, iron (a transition element) in the myoglobin is released and causes free radical formation in proximal tubule cells of the nephron and lipid peroxidation (9,10). Studies conducted in recent years have determined that NO and free radicals play an important role in the pathophysiology of MAKI. Administration of antioxidant agents against free radical damage is reported to have protective effects (9).

Nitric oxide synthesises L-arginine from amino acids as a result of a range of reactions catalysed by NO synthase (NOS) enzymes. L-arginine has a role in the control of glomerular hemodynamics in kidneys, the tubuloglomerular feedback mechanism, secretion of renin and the regulation of water and Na+ (11,12). The reduction in kidney NO production in MAKI possibly increases renal injury (8). Three isoforms of NOS have been identified: neuronal NOS (nNOS), inducible NOS (iNOS) and endothelial NOS (eNOS). The literature reports that all these isoforms are expressed in the kidneys. The NO necessary for physiological functions is synthesised by eNOS and nNOS. iNOS causes longer duration and higher amounts of NO synthesis compared to the other isoforms, which intensifies the physiological effects of NO. Studies have reported that, while iNOS activity increased in the experimental MAKI model, eNOS activity is reduced (13).

Baicalin is used for the treatment of different diseases in Chinese medicine and is obtained from the roots of the *Scutellaria baicalensis* plant. Laboratory studies of baicalin demonstrate bactericidal, anti-inflammatory and antioxidant effects. It also reduces NO production by suppressing iNOS activity (14,17). Baicalin has shown protective treatment effects in many studies, including experimental renal injury models (14,15,18). According to our research, there is no study investigating the effect of baicalin on experimental MAKI.

Baicalin can modulate NO production and inhibit oxidative stress. Therefore, we hypothesised that baicalin has protective effects via the inhibition of free radical production in the experimental MAKI model induced by administering hypertonic glycerol. We aimed to investigate the effects of baicalin on renal injury, NO metabolism, histopathological changes and renal functions.

## MATERIALS AND METHODS

In this study, we used Sprague Dawley male rats weighing 150-200 g raised in the Experimental Animals Production and Research Laboratory of Trakya and kept under standard laboratory conditions (22±1 °C, 12-hour light/dark cycle). Rats in the Experimental Animals Laboratory were given standard rat feed and tap water. Permission was obtained for the study from Trakya University Animal Experiments Local Ethics Committee (2012/33).

### Experimental design

**Figure 1:**
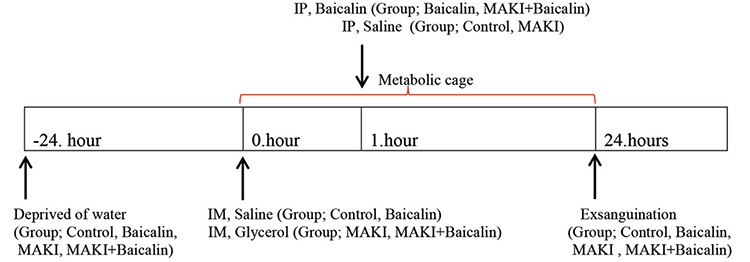
Experimental design, Baicalin was injected 1 hour after glycerol injection.

The male Sprague Dawley rats were randomly divided into 4 groups. Control (n=8), baicalin (n=8), MAKI (n=10) and MAKI + baicalin (n=10). The rats were deprived of water for 24 hours before receiving intramuscular injection. In the control and baicalin groups, a saline solution (8 ml/kg) was injected intramuscularly (8 ml/kg), and the MAKI and MAKI + baicalin groups were given 50% glycerol (8 ml/kg). All intramuscular injections were applied in equal volumes for all hind limbs. The rats were placed in individual metabolic cages after the glycerol injection for 24 hours for urine collection. One hour after intramuscular injection of saline/glycerol, the control and MAKI groups received saline, and the baicalin and MAKI + baicalin groups were given 200 mg/kg baicalin intraperitoneally. All groups received the same volume of intraperitoneally injections. The experimental design is shown in (Figure 1).

After administration of 10 mg/kg xylazine and 50 mg/kg ketamine anaesthesia, the animals were sacrificed and both kidneys obtained. Half of the right kidney was placed in 10% formalin solution for histopathological investigation, the other half and all of the left kidney were stored at -80 °C for MDA, GSH and nitrite-nitrate determinations. Blood and urine samples were centrifuged at 1500 × g for 10 minutes at 4 °C and then stored at -80 °C.

### Biochemical study

Serum urea, creatinine, sodium (Na+), potassium (K+) levels; alanine aminotransferase (ALT), aspartate aminotransferase (AST) and creatine kinase (CK) activities; and urine creatinine and sodium measurements were performed on an autoanalyser (Konelab Prime 60i, Thermo Scientific, Finland) in Trakya University Biochemistry Laboratory.

### Histological study

For light microscope investigation, kidneys were cut in the sagittal plane and fixed in 10% formalin, submerged in paraffin blocks and cut to sections of 5 µm thickness. Sections were stained with the haematoxylin-eosin method for light microscopy. Preparations were semi-quantitatively assessed for glomerular necrosis, glomerular basal membrane thickening, mesangial matrix widening, tubular hydropic degeneration and tubular dilatation. Pathological changes are expressed as follows: 0, no pathology; +1, focal; +2, moderate focal; +3, multifocal and +4, diffuse (19,20).

### Immunohistochemical study

Labelled polyclonal or monoclonal antibodies were used against the investigated proteins. Tissue blocks were fixed in 10% formalin and submerged in paraffin. They were sliced to 4 µm thickness. Sections were left for 1 night at 37 °C and deparaffinised with xylol twice for 15 minutes each. The sections were then passed through an alcohol series beginning with pure alcohol and then 90-80-70% and finally washed with distilled water, completing deparaffinisation process. In the next step, the sections were heated 4 times in a microwave oven for 5 minutes in buffer solution (citrate buffer) and left at room temperature to cool down. Then the sections were washed with distilled water and dipped in Tris buffer solution.

To reduce non-specific background staining due to endogenous peroxide, the sections were treated for 20 minutes with 3% hydrogen peroxide (H_2_O_2_). The sections were washed with distilled water, then again dipped in Tris buffer solution. With the aim of preventing non-specific background staining, ultra V-block (protein blockage) was applied at room temperature for 5 minutes. Then the sections were incubated for 30 minutes with inducible nitric oxide synthase (iNOS, GeneTex, GTX 15326) and endothelial nitric oxide synthase (eNOS, GeneTex, GTX 15326) primary antibodies. After washing 3 times with Tris buffer, the sections were incubated with biotinylated secondary antibodies for 20 minutes and enzyme-labelled streptavidin for 20 minutes.

The sections taken from Tris buffer were left in 3-amino-9-ethylcarbazole (AEC) chromogen for 20 minutes and washed with distilled water. Then, they were incubated with Mayer’s hematoxylin for 3 minutes for inverse staining. The sections were washed with flowing water and then sealed with aqueous mount gel. The sections prepared for each case were investigated under a light microscope.

To check the specificity of immune staining for a positive control, kidney sections were used in accordance with the antibody instructions by producer. Cases were assessed in terms of cytoplasmic staining prevalence and intensity. Staining prevalence was taken as a percentage of epithelial component for each case. Staining intensity reflects cytoplasmic staining on tissue sections and chromogen staining of cells with scoring as follows: 0, no staining; +1, mild staining; +2, moderate intensity staining and +3, strong staining. Total immunohistochemical scoring was obtained by multiplying the staining prevalence and intensity with scores varying from 0 to 300 (21).

### Renal tissue homogenisation

For GSH and MDA, 0.15 M KCl solution was used, and 50 mM phosphate buffer (pH 7.4) was used for NO levels prepared at 10% (w/v). Homogenates were centrifuged at 2608xg for 10 minutes at 4 °C, and then the supernatant was separated. The supernatants were used for spectrophotometric measurements of MDA, NO and GSH.

### Determination of malondialdehyde amount

The final product of lipid peroxidation of MDA enters a reaction with thiobarbituric acid (TBA) in a hot, acidic environment. The resulting pink colour was measured spectrophotometrically (22,23). The results are given as MDA nmol/g wet tissue. Tissue homogenates (0.2 ml) diluted 10 times were mixed with 0.2 ml of 8.1% SDS, 1.5 ml of 20% acetic acid, 1.5 ml of 0.8% TBA and 0.6 ml distilled water. The mixture was left in hot water bath for 1 hour at 95 °C. After cooling under tap water, it was centrifuged at 2608xg for 10 minutes. Absorbance was read with a spectrophotometer at 650 nm against a homogenate-free blind reagent. A consumption coefficient of 1.56x105 M-1 cm-1 was used to calculate MDA and was determined as tissue nmol MDA per gram.

### Measurement of glutathione levels

The Ellman’s reagent was used to determine the glutathione content based on spectrophotometric identification of the colour formed by free sulfhydryl groups in tissue homogenates (24). GSH concentration was read at 412 nm with a spectrophotometer. GSH levels were calculated using an extinction coefficient (∑=1.36 104 M-1 cm-1). The results are given as µmol GSH/g tissue.

### Determination of nitrite and nitrate

Nitrite and nitrate are primary oxidation products forming after the reaction of nitric oxide with oxygen. As a result, concentration of nitrite/nitrate in serum was used as a marker of NO synthesis.

Determination of the nitrite and nitrate amount was based on the Griess reaction (25). In this reaction, nitrite reacts with a mixture of N-(1-Naphthyl)ethylenediamine and sulphanilamide and forms a chromophore that has strong absorbance at 545 nm.

Samples were deproteinised with Somogyi reagent. Nitrate was reduced to nitrite with copper-coated cadmium in glycine buffer (pH 9.7). Total nitrite/nitrate concentration was calculated using sodium nitrate standard solution. Results are stated as mmol/L for serum and urine and mmol/mg protein for tissue.

### Statistical analysis

The results were expressed as mean ± standard deviation. The normal distribution of variables was determined using the one sample Kolmogorov-Smirnov test. The Kruskal-Wallis test was used for comparison of numeric variables among groups, and then the Mann-Whitney U test with Bonferroni correction was used for multiple comparisons when a significant difference was obtained among groups. For the Kruskal-Wallis test, p<0.05 was considered as statistically significant. Additionally, p<0.017 was set for Mann-Whitney U tests with Bonferroni correction in order to prevent the increment of type 1 error. Statistical analyses were performed using SPSS 20.0 (IBM SPSS Inc., Chicago, IL, USA) statistical software.

## RESULTS

### Biochemical results

In our study, there were significant differences in serum AST (p<0.001), ALT (p<0.001), CK (p<0.001), urea (p<0.001), creatinine (p<0.001) potassium (p<0.001) and NO (p=0.001) levels between the control and MAKI groups. There were also significant increases in the kidney NO levels (p=0.006), MDA (p<0.001) and fractional sodium excretion (p=0.001) between the control and MAKI group. There were statistically significant decreases in urine creatinine (p=0.001), NO (p=0.005) and creatinine clearance (p=0.002) levels in the same groups.

There was a statistically significant decrease in renal GSH (p=0.001) and NO (p=0.001) levels between the MAKI and MAKI + baicalin groups. The biochemical parameters of the study groups are shown in (Table 1).

### Histopathological results

**Figure 2a-d:**
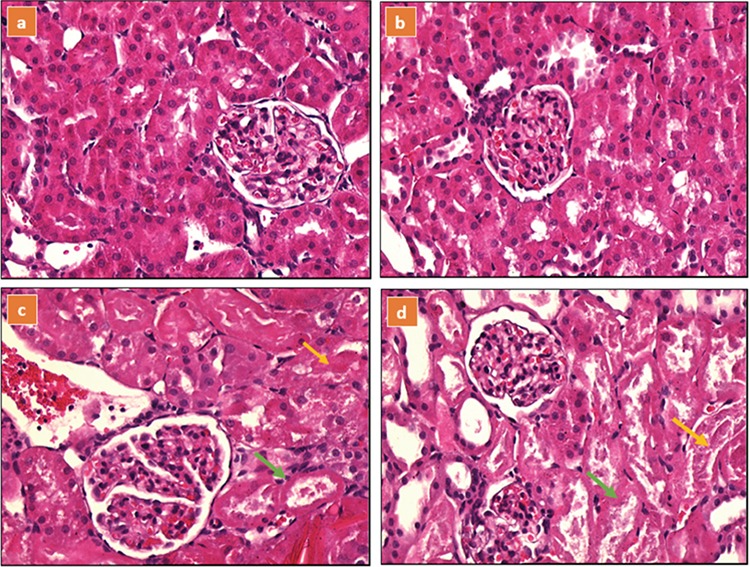
Haematoxylin and eosin stained sections of rat kidneys, (a) control group; (b) baicalin group; (c) MAKI group; kidney section of a glycerol-treated rat showing moderate necrosis and cast formation; (d) MAKI + baicalin group; kidney section of a glycerol + baicalin treated rat showing moderate necrosis and cast formation. Extensive necrosis (green arrow) and cyst formation in the lumen (yellow arrow) were observed in the tubular epithelium in MAKI and MAKI + baicalin groups.

When the HE-stained kidney sections of rats in the control and baicalin groups were examined with a light microscope, glomeruli and tubules appeared to be normal on microscopic sections with no necrosis or cast formation. When the HE-stained kidney sections of rats in the MAKI and MAKI + baicalin groups were investigated, necrosis was encountered in the tubular epithelium. Accumulation of dense proteinous material in the form of casts was observed beginning in the subcortical area of the proximal, distal and collector tubule lumens and extending to the renal pelvis. There was clear dilatation of the tubules. In distal tubular cells, hydropic cytoplasmic swelling and vacuolisation were noted. Apart from clear congestion of the glomerulus, no change was observed. There was oedema of the peritubular stroma and congestion of the vascular structures and signs of regeneration-like nuclear hypertrophy and nucleoli manifestation in tubular epithelial cells. Histopathological findings for all the groups are shown in (Table 2, Figure 2).

### Immunohistochemical assessment

Immunohistochemical investigation of renal tissue assessed iNOS and eNOS and prevalence and intensity of cytoplasmic staining. Staining prevalence was based on the percentage of epithelial component. No staining was scored as 0, <25% was +1, 25-50% was +2, 50-75% was +3 and >75% was +4. Staining intensity was scored as 0 in cases with no staining observed, +1 for cases with slight staining, +2 for moderate staining and +3 for cases with strong staining. Total immunohistochemical scoring was obtained by multiplying the staining prevalence and intensity scores.

**Figure 3a-d:**
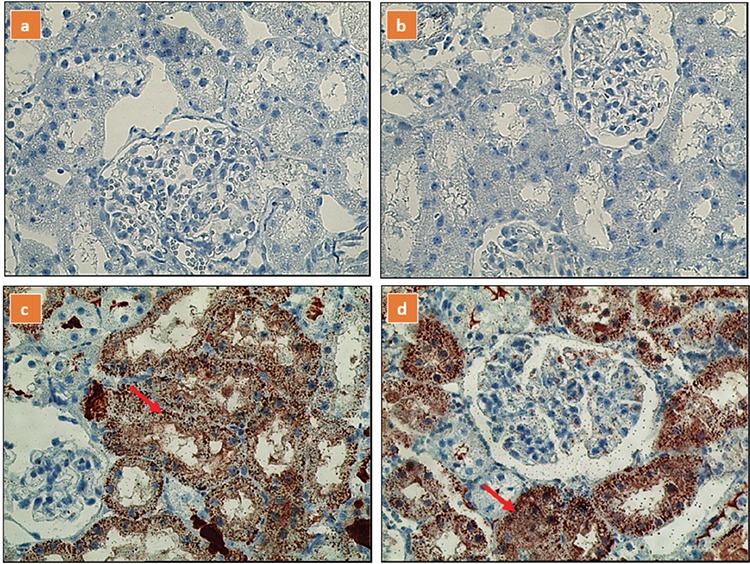
Glomerular immunostaining for endothelial nitric oxide synthase in different groups, (a) control group; (b) baicalin group; (c) MAKI group; (d) MAKI + baicalin group. In the MAKI and MAKI + baicalin groups, severe and diffuse staining (red arrow) was observed.

**Figure 4a-d:**
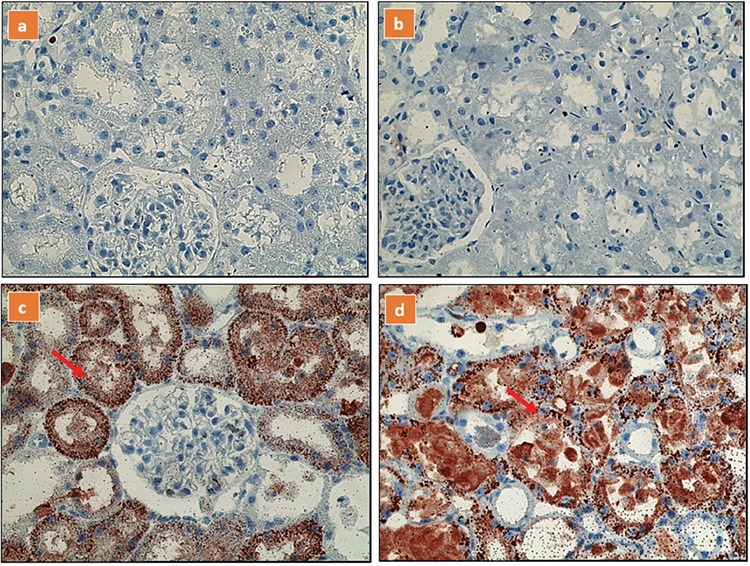
Glomerular immunostaining for inducible nitric oxide synthase in different groups, (a) control group; (b) baicalin group; (c) MAKI group; (d) MAKI + baicalin group. In the MAKI and MAKI + baicalin groups, severe and diffuse staining (red arrow) was observed.

No reaction was observed in immunohistochemical staining with iNOS and eNOS in the distal tubular epithelial cells of control and baicalin groups. In the MAKI and MAKI + baicalin groups, severe and diffuse staining was observed. The immunohistochemical findings of all groups are shown in (Table 3, Figure 3, 4).

## DISCUSSION

In the MAKI model, (induced by administering 50% glycerol to rats) we observed an elevation of MDA, NO levels, the kidney function markers of serum urea and serum creatinine, AST, ALT, CK and serum K+ levels and a reduction in the glomerular filtration marker of creatinine clearance and a statistically significant increase in the tubular function marker of fractional sodium excretion. When histopathological sections were investigated, necrosis and cast formation in the tubular lumen were observed. Additionally, iNOS and eNOS activities increased in groups with renal failure; however, treatment with baicalin did not have a beneficial effect. Other experimental models indicate the protective effects of baicalin, but in this study, no protective effects on lipid peroxidation, renal function and histopathological changes were observed in the MAKI group.

Free radicals are known to play an important role in the pathogenesis of different renal diseases, including MAKI. Levels of MDA, a marker of free radical damage in the kidney and the final product of lipid peroxidation, were reported to be significantly increased in previous studies (26). The increase in MDA levels shows excess radical formation that exceeds the antioxidant enzymes in the kidney. In our study, as in previous studies, a significant increase in MDA levels was observed in rat kidneys (6,7). However, interestingly enough, GSH levels, functioning through glutathione and a marker of antioxidant defence, did not significantly change in the MAKI group. Kaliman et al. (27) reported that in the kidney, the GSH levels were significantly reduced 2 hours after glycerol injection in the MAKI group compared to the control group. However, this increase was not significant at hours 6 and 24. The GSH levels investigated in this study were similar to the results of our study.

In our study, the increase in serum urea and creatinine levels (an indicator of glomerular dysfunction) and the decrease in creatinine clearance were compatible with the results of previous studies (25,26,28,29,30,31). The increase in fractional sodium excretion showing disrupted tubular function was similar to previous study results (32). 

In conclusion, the biochemical and histological findings from rats in the MAKI group showed a clear degree of AKI development.

In the MAKI group, a significant increase occurred in NO levels in kidney tissue. NO plays a role in many physiologic and pathologic events while simultaneously acting like a free radical. NO increases during kidney injury, and large amounts of molecular oxygen are transported to the tissues, resulting in a large amount of free oxygen and nitrogen radicals (33).

In the kidney tubules, increased iNOS stimulated by cytokines in the proximal tubules specifically indicates that the changes we observed may be related to NO. In our study, immunohistochemical staining in the MAKI group showed increased iNOS and eNOS positive cell numbers in glomerular clusters. The increase in iNOS and eNOS activity is reported to be in parallel with increased nitrite and nitrate within the glomerulus (34). In the MAKI and MAKI + baicalin group, a reduction in NO levels was observed. These results were in accordance with the previous study performed in the same model (35).

In our study, we believe injury in the MAKI group was the result of increased peroxynitrite forming linked to the increase in iNOS activity. Under normal conditions, the NOS enzyme forms NO from arginine. When either arginine or tetrahydrobiopterin levels are insufficient, the NOS produces a superoxide radical instead of NO. This event is reported to cause uncoupling of NOS (36).

In this situation, electrons moving from the reductase region to the oxidase region of the NOS enzyme become attracted to molecular oxygen instead of arginine (37). In our study, the increase in eNOS activity may be the result of eNOS uncoupling. Liu et al. (35) investigated iNOS and eNOS activity and NO levels administered with glycerol and reported that nitrate level and iNOS activity increased in the group with induced MAKI, while eNOS activity reduced. The eNOS activity in Liu et al. (35) study does not comply with the eNOS activity obtained in our study, possibly due to the fact that this study investigated eNOS activity 7 days after the glycerol injection.

In our study, glycerol was administered intramuscularly to develop MAKI, and in the MAKI + baicalin group 1 hour after intraperitoneally glycerol administration, there was no significant difference in MDA, serum urea, creatinine, serum K+, and serum NO levels, ALT, AST, CK enzyme activities, urine NO and urine creatinine and creatinine clearance, and fractional sodium excretion compared to the MAKI group. However, in the MAKI + baicalin group a statistically significant reduction was observed when GSH and kidney NO levels were compared with the MAKI group. In the MAKI + baicalin group, when HE-stained kidney sections were examined with light microscope, the histological characteristics were similar to the MAKI group.

Many in vitro and in vivo studies demonstrate that baicalin protects certain organs such as the kidney, liver and pancreas from immune system damage and inflammatory mediators. A recent study investigated the effects of baicalin on ischaemia-reperfusion (I/R) injury. This study found that baicalin inhibited the proinflammatory cytokine transcription factor of NF-κB in I/R injury and reduced oxidative damage (38).

The lack of compliance between our study results and the results of previous studies may be linked to differences in baicalin administration, administration time and duration, and different effective factors in the formation mechanism of experimental MAKI in other experimental models. Additionally, the effects of baicalin may be observed after long-term administration. Zhang et al. (17) reported a strong treatment effect linked to high dose and more than 1 injection due to the short half-life of baicalin. In our study, baicalin was administered in a single dose which might have lead to a reduction in observed protective effects.

When all these findings and literature results are evaluated together, our study remains insufficient to definitively show the positive effect of baicalin on experimental MAKI. We believe that more comprehensive studies might be need to design investigation of the net effects of baicalin, including dose, administration way, time and duration of the drug, along with histopathological variations by electron microscope and studies in molecular levels.

## Figures and Tables

**Table 1:**
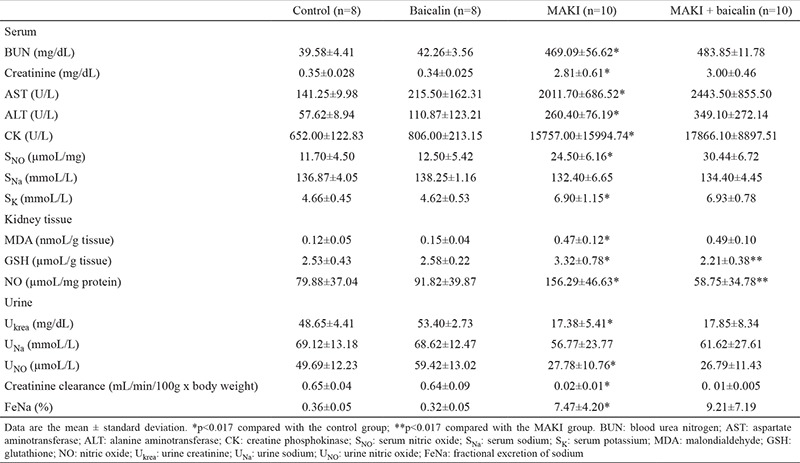
Biochemical results of experimental groups

**Table 2:**
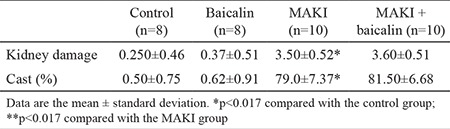
Histological findings

**Table 3:**
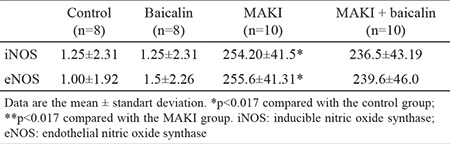
Immunohistochemical staining intensity and score for iNOS and eNOS in the kidney specimens of rats in all experimental groups

## References

[1] Glassock RJ, Massry SG, Humes In:, editors.. -77., Glassock RJ, Massry SG (2001). Diagnosis, clinical presentation, and management. Massry And Glassock’s The Textbook of Nephrology. 4 ed.

[2] Sever MS, Vanholder R, RDRTF of ISN Work Group on Recommendations for the Management of Crush Victims in Mass Disasters (2012). Recommendation for the management of crush victims in mass disasters. Nephrol Dial Transplant.

[3] Abul-Ezz SR, Walker PD, Shah SV (1991). Role of glutathione in an animal model of myoglobinuric acute renal failure. Proc Natl Acad Sci USA.

[4] Shah SV, Walker PD (1988). Evidence suggesting a role for hydroxyl radical in glycerol-induced acute renal failure. Am J Physiol.

[5] Sever MS, Erek E, Vanholder R, Koc M, Yavuz M, Aysuna N, et al (2004). Lessons learned from the catastrophic Marmara earthquake: factors influencing the final outcome of renal victims. Clin Nephrol.

[6] Aydogdu N, Atmaca G, Yalcin O, Batcioglu K, Kaymak K (2004). Effects of caffeic acid phenethyl ester on glycerol-induced acute renal failure in rats. Clin Exp Pharmacol Physiol.

[7] Aydogdu N, Atmaca G, Yalcin O, Taskiran R, Tastekin E, Kaymak K (2006). Protective effects of L-carnitine on myoglobinuric acute renal failure in rats. Clin Exp Pharmacol Physiol.

[8] Zager RA (1996). Rhabdomyolysis and myohemoglobinuric acute renal failure. Kidney Int.

[9] Vanholder R, Sever MS, Erek E, Lameire N (2000). Rhabdomyolysis. J Am Soc Nephrol.

[10] Chander V, Chopra K (2005). Molsidomine, a nitric oxide donor and L-arginine protects against rhabdomyolysis-induced myoglobinuric acute renal failure. Biochim Biophys Acta.

[11] Baylis C (2006). Arginine, arginine analogs and nitric oxide production in chronic kidney disease. Nat Clin Pract Nephrol.

[12] Palm F, Teerlink T, Hansell P (2009). Nitric oxide and kidney oxygenation. Curr Opin Nephrol Hypertens.

[13] Eghbalzadeh K, Brixius K, Bloch W, Brinkmann C (2014). Skeletal muscle nitric oxide (NO) synthases and NO-signaling in "diabesity" what about the relevance of exercise training interventions?. Nitric Oxide.

[14] Kim SJ, Moon YJ, Lee SM (2010). Protective effects of baicalin against ischemia/reperfusion injury in rat liver. J Nat Prod.

[15] Rabb H, Ramirez G, Saba SR, Reynolds D, Xu J, Flavell R, et al (1996). Renal ischemic-reperfusion injury in L-selectin-deficient mice. Am J Physiol.

[16] Cai X, Li C, Du G, Cao Z (2008). Protective effects of baicalin on ligature-induced periodontitis in rats. J Periodontal Res.

[17] Zhang XP, Tian H, Lai YH, Chen L, Zhang L, Cheng QH, et al (2007). Protective effects and mechanisms of Baicalin and octreotide on renal injury of rats with severe acute pancreatitis. World J Gastroenterol.

[18] Zhang Z, Gao X, Guo M, Jiang H, Cao Y, Zhang N (2017). The Protective Effect of Baicalin Against Lead-Induced Renal Oxidative Damage in Mice. Biol Trace Elem Res.

[19] Rabb H, Mendiola CC, Dietz J, Saba SR, Issekutz TB, Abanilla F, et al (1994). Role of CD11a and CD11b in ischemic acute renal failure in rats. Am J Physiol.

[20] no authors (2008). B. A. Streptozosin ile diyabet oluşturulan sıçanlarda pirolidyum dithiyokarbamat’ın böbrek dokusu üzerine koruyucu etkisi. İstanbul: T.C. Sağlık Bakanlığı Bakırköy Dr. Sadi Konuk Eğitim ve Araştırma Hastanesi.

[21] Korkmaz A, Kolankaya D (2013). Inhibiting inducible nitric oxide synthase with rutin reduces renal ischemia/reperfusion injury. Can J Surg.

[22] Ohkawa H, Ohishi N, Yagi K (1979). Assay for lipid peroxides in animal tissues by thiobarbituric acid reaction. Anal Biochem.

[23] Devi GS, Prasad MH, Saraswathi I, Raghu D, Rao DN, Reddy PP (2000). Free radicals antioxidant enzymes and lipid peroxidation in different types of leukemias. Clin Chim Acta.

[24] Zakowski JJ, Tappel AL (1978). A semiautomated system for measurement of glutathione in the assay of glutathione peroxidase. Anal Biochem.

[25] Cortas NK, Wakid NW (1990). Determination of inorganic nitrate in serum and urine by a kinetic cadmium-reduction method. Clin Chem.

[26] Beckman JS, Koppenol WH (1996). Nitric oxide, superoxide, and peroxynitrite: the good, the bad, and ugly. Am J Physiol.

[27] Kaliman PA, Strel'chenko EV, Nikitchenko IV, Filimonenko VP (2003). Heme oxygenase activity and some indices of antioxidant protection in rat liver and kidney in glycerol model of rhabdomyolysis. Bull Exp Biol Med.

[28] Aydogdu N, Atmaca G, Yalcin O, Batcioglu K, Kaymak K (2004). Effects of caffeic acid phenethyl ester on glycerol-induced acute renal failure in rats. Clin Exp Pharmacol Physiol.

[29] Aydogdu N, Atmaca G, Yalcin O, Batcioglu K, Kaymak K (2004). Effects of exogenous melatonin on myoglobinuric acute renal failure in the rats. Ren Fail.

[30] Ustundag S, Sen S, Yalcin O, Ciftci S, Demirkan B, Ture M (2009). L-Carnitine ameliorates glycerol-induced myoglobinuric acute renal failure in rats. Ren Fail.

[31] Boutaud O, Moore KP, Reeder BJ, Harry D, Howie AJ, Wang S, et al (2010). Acetaminophen inhibits hemoprotein-catalyzed lipid peroxidation and attenuates rhabdomyolysis-induced renal failure. Proc Natl Acad Sci USA.

[32] Korrapati MC, Shaner BE, Schnellmann RG (2012). Recovery from glycerol-induced acute kidney injury is accelerated by suramin. J Pharmacol Exp Ther.

[33] Ronson RS, Nakamura M, Vinten-Johansen J (1999). The cardiovascular effects and implications of peroxynitrite. Cardiovasc Res.

[34] Dunlop M (2000). Aldose reductase and the role of the polyol pathway in diabetic nephropathy. Kidney Int Suppl.

[35] Liu Y, Fu X, Gou L, Li S, Lan N, Zheng Y, et al (2013). L-citrulline protects against glycerol-induced acute renal failure in rats. Ren Fail.

[36] Pall ML (2007). Nitric oxide synthase partial uncoupling as a key switching mechanism for the NO/ONOO- cycle. Med Hypotheses.

[37] Kim JH, Bugaj LJ, Oh YJ, Bivalacqua TJ, Ryoo S, Soucy KG, et al (2009). Arginase inhibition restores NOS coupling and reverses endothelial dysfunction and vascular stiffness in old rats. J Appl Physiol (1985).

[38] Lin M, Li L, Li L, Pokhrel G, Qi G, Rong R, et al (2014). The protective effect of baicalin against renal ischemia-reperfusion injury through inhibition of inflammation and apoptosis. BMC Complement Altern Med.

